# Dr. Frederick Walter Smith

**Published:** 1917-10

**Authors:** 


**Dr. Frederick Walter Smith,** N. Y. University 1873, died at his home in Syracuse of cerebral haemorrhage, July 31, aged 58. He was Obstetrician to St. Joseph's Hospital and Health

Officer of Syracuse since 1902, having previously had the title of Health Commissioner, 1891-97. He was on the State Board of Health from 1895 to 1901 and had been coroner of Onondaga Co. for several years.

